# Does club convergence matter in health outcomes? Evidence from Indian states

**DOI:** 10.1186/s12889-023-16972-2

**Published:** 2023-11-03

**Authors:** Ajit Nag, Andrej Privara, Beata Gavurova, Jalandhar Pradhan

**Affiliations:** 1 Department of Humanities and Social Sciences National Institute of Technology, Rourkela, Odisha India; 2 Faculty of National Economy University of Economics in Bratislava, Bratislava, Slovak Republic; 3 Center for Applied Economic Research, Faculty of Management and Economics, Tomas Bata University in Zlin, Zlín, Czech Republic

**Keywords:** Club convergence, Health status, Kernel density, Indian states

## Abstract

**Background:**

Population health is vital to a nation’s overall well-being and development. To achieve sustainable human development, a reduction in health inequalities and an increase in interstate convergence in health indicators is necessary. Evaluation of the convergence patterns can aid the government in monitoring the health progress across the Indian states. This study investigates the progressive changes in the convergence and divergence patterns in health status across major states of India from 1990 to 2018.

**Methods:**

Sigma plots (*σ*), kernel density plots, and log t-test methods are used to test the convergence, divergence, and club convergence patterns in the health indicators at the state level.

**Results:**

The result of the sigma convergence suggests that life expectancy at birth has converged across all states. After 2006, however, the infant mortality rate, neonatal mortality rate, and total fertility rate experienced a divergence pattern. The study’s findings indicate that life expectancy at birth converges in the same direction across all states, falling into the same club (Club One). However, considerable cross-state variations and evidence of clubs’ convergence and divergence are observed in the domains of infant mortality rate, neonatal death rate, and total fertility rate. As suggested by the kernel density estimates, life expectancy at birth stratifies, polarizes, and becomes unimodal over time, although with a single stable state. A bimodal distribution was found for infant, neonatal, and total fertility rates.

**Conclusions:**

Therefore, healthcare strategies must consider each club’s transition path while focusing on divergence states to reduce health variations and improve health outcomes for each group of individuals.

## Introduction

Life expectancy, child mortality, and total fertility rate are essential elements of human health and vital indicators of economic, social, medical, and technological advancements [1–7], which reflect the economic development and social well-being, disease rates, environmental quality, and technological advancement in a country [6, 8, 9]. A country’s significant successes include raising life expectancy and lowering child mortality and total fertility rate [6, 10, 11]. Apart from enhanced individual life expectancy, premature mortality has significantly reduced across age categories worldwide [12–15]. Over the last two decades, health status has substantially improved worldwide. However, population health could still be a severe problem in developing countries [16]. Achieving equity in health outcomes across countries is a salient feature of global development [17–19].

In recent years, there has been a growing acknowledgment of the increasing significance of convergence, not just in terms of income but also concerning health and health transition. Researchers have explored convergence and divergence hypotheses in global health trends as nations move towards closer economic integration and market unification [20–22]. Over the past two centuries, global life expectancy at birth has more than doubled, rising from 39 years to over 85 years [23, 24]. However, there are still marked disparities between developed and developing countries. Developed countries have an average life expectancy of above 80 years and low child mortality rates (below five deaths/1000 live births) [25]. In contrast, low-middle-income countries have average life expectancies below 70 years and higher child mortality rates (around 30 deaths/1000 live births) [25]. Bridging these gaps in health outcomes among nations and different age groups is a crucial concern in global development. Therefore, it is crucial to deeply understand this theory by assessing convergence within a multi-input, multi-output framework, especially in development sectors [26]. Most studies have focused on comparing developed and developing nations in studying the convergence of health issues across populations. Nevertheless, it neglects the context of emerging countries whose development paths still need clarification [27–29].

Although global health has improved, preventing morbidity and mortality requires urgent attention [30, 31]. Child and infant mortality indicators have improved gradually in India over the period. However, the rates remain concerning compared to developing countries with similar socio-economic status [32–34]. Although significant strides have been made in reducing infant and child mortality, the persistent disparities between states and regions within India remain a vital concern [35, 36]. Uneven growth and inconsistent patterns in child mortality reduction over the past decades highlight the challenging nature of achieving sustainable development in the country [36]. Achieving health and well-being requires eliminating health disparities.

The total fertility rate is a significant determinant of population growth [16]. Analyzing fertility rate disparities across different regions becomes essential to comprehend recent shifts in population dynamics in emerging countries [37]. Excessive population growth strongly affects food production, the environment, biodiversity, and a country’s economy [38–41]. During the past decade, there has been a convergence in fertility trends among developed nations [28, 42]. A recent study by Bongaarts and Hodgson [43] explored the levels and patterns of fertility in 97 developing nations from 1950 to 2020. The study revealed that certain developing countries have already experienced the fertility transition; while most countries are currently undergoing this transition, some have just begun to witness declining fertility rates. However, it is essential to note that global fertility rates have converged over time, albeit with substantial variations between different countries [27]. Convergence occurs when the difference between country or state variations declines [27, 44]. Future international and national planning depends on understanding the reasons for this remarkable population growth [45]. Based on historical and current TFR data [46], the country’s TFR levels will converge at 2.1 replacement levels during the decades up to 2100. India, with its large population, varied topography, and swift changes in fertility rates, offers an excellent opportunity to explore convergence theory. Although the latest National Family Health Survey-5 (NFHS-5) data indicate a significant decline in India’s fertility rate to 2.1, this reduction is not uniform across all states [47].

The persistence of health inequality across the globe has remained a neglected aspect of health disparity research. Several studies have highlighted the disparity in health among the advanced and lagged regions, and India is no exception [48–51]. India’s health transition can be attributed to factors like sizable population size, poverty, mortality, and inequality in health [49, 50, 52–54]. Therefore, increased efficiency concerning achieving equity and improved regional public health outcomes play a vital role in India's health transition process. The Lancet Commission has launched a highly ambitious framework to achieve a grand convergence in health within a generation by 2035 [55].

Previous research has shown varying opinions on whether the convergence of population health is linked to overall improvements in health outcomes worldwide. A recent study by Aksan & Chakraborty [56] analysed changes in global life expectancies from 1960 to 2015 and found that while life expectancy at birth has become similar across countries, there is now more significant variation in late-life longevity. The study showed that differences in healthcare access, influenced by income inequality, have contributed to the divergence in survival gains among the elderly. In the Indian context, using both standard and cutting-edge convergence metrics, Goli & Arokiasamy [54] analysed the convergence hypothesis for health and health inequalities in major Indian states. They discovered that there is convergence in life expectancy at birth, child immunization, and underweight rates, but also that from the 1990s, convergence was increasingly being replaced by divergence. Similarly, Siddiqui et al. [51] also examined health inequalities across major Indian states using the same methodology. They found that the absolute β-convergence measure showed convergence in life expectancy at birth among the states. The β- and σ-convergence results showed that post-2000, convergence replaced divergence for child and maternal mortality indicators. Furthermore, applying the log t-test has been limited to a few studies that have explored and identified the existence of club convergence in HDIs among Indian states [57].

Most studies that have examined health indicators such as life expectancy, infant survival, and total fertility rate across various states of India have used beta (β) and sigma (σ) convergence analyses. However, a few studies have used the Phillips and Sul [58] methodology to evaluate these health outcome variables at the state level in India. This study aims to analyze the changes in the patterns of convergence and divergence in health status across major states of India from 1990 to 2018. Additionally, we also try to identify the existence of club convergence by employing the method suggested by Phillips and Sul [58] and Kernel density estimators.

## Methodological framework

The analysis is based on the following methodology to examine the variations in health improvement and identify club convergence. Phillips and Sul [58] developed a framework Log *T*-test to test the convergence hypothesis. According to this theory, hypothesis rejection indicates a convergence across the states regarding selected health indicators. This convergence is called ‘club convergence’ when it occurs among a subset of states. In economics, the concept of “club convergence” pertains to a phenomenon in which distinct groups or subsets of economies, frequently denoting regions, states, or countries exhibiting comparable characteristics or policies, tend to converge in terms of their economic performance and outcomes [58–61]. Similarly, health economics suggests distinct groups of regions or countries following different trajectories in health indicators based on their initial health status. The methodology proposed by Phillips and Sul [58] differs from the traditional *β*-convergence and *σ*-convergence analysis introduced by Barro & Sala-I-Martin [62, 63], who suggest that there are two types of convergence namely, *β*-convergence and *σ*-convergence. Absolute *β*-convergence refers to the process in which lagged regions progress faster than the advanced regions [62, 64] and catch up ultimately [65–67]. Conversely, the sigma convergence estimates show the variation status in reducing the cross-sectional dispersion of a variable over time [62].

The discussion on the convergence hypothesis originated from the neoclassical growth theory developed by Solow & Swan [68, 69]. Inada [70] highlighted that Solow’s critical assumption is that when the marginal product of capital or labor approaches infinity, capital or labor touches zero and vice versa. Moreover, the concept of *β* and *σ* convergence was first introduced by Baumol [71]. The latest methodology related to the convergence theory proposed by Phillips and Sul [58, 59] is based on a general nonlinear time-varying factor model, which has become popular in convergence analysis.

### Sigma convergence

First, this paper focused on sigma convergence to examine changes in the patterns of convergence and divergence in health status across major states in India. Sigma convergence provides an intuitive understanding of convergence by measuring the reduction in cross-sectional dispersion of a variable over time [62]. It illustrates the intermittent dispersion evolution over time through a sigma convergence approach by the standard deviation and coefficient of variation as an inquiry. In this paper, we used standard deviation and the coefficient of variation (CV) to indicate sigma convergence. The σ -convergence model is expressed mathematically as follows:

1 $$\sigma ={\sigma }_{t}>{\sigma }_{t+T}$$

where ${\sigma }_{t}$ is the indicators’ standard deviation at time t. If the parameter ${\sigma }_{t+T}$ decreases with time, convergence is implied; otherwise, divergence is implied [62, 63, 72]. The CV is calculated using the following equation to show the cross-sectional dispersion in the selected outcome variables [73, 74].

2 $${CV}_{t}=\sqrt{\frac{1}{N}} \sum_{i=1}^{N}{\left(\frac{{X}_{i,t}-{\overline{X} }_{t}}{\overline{{X }_{t}}}\right)}^{2} ,{\overline{X} }_{t}=\frac{1}{N}\sum_{i=1}^{N}{X}_{it} for t=\mathrm{1,2},3,\dots ..,T$$

Where ${X}_{i,t}$ represents the selected health variable in this paper, and N and T represent the number of states and years, respectively.

### Log t-test convergence

Phillips and Sul [58] (hereafter, PS) approach, often known as the “log T-test,” was used to analyze the possibility of convergence, club convergence, and divergence in the selected health indicators across the major Indian states. The PS model may be characterized as a nonlinear time-varying factor model. For a better understanding, consider the following equation:

3 $${X}_{it}={\mathrm{g}}_{it}{\mu }_{t}+{a}_{it}$$

where ${X}_{it}$ is a measure health status such as LEB, IMR, NNMR, and TFR observed across i = 1,..., N and t = 1,..., T, which denote the number of Indian states and sample size, respectively. ${X}_{it}$ is frequently decomposed into two components: ${\mathrm{g}}_{it}$, the idiosyncratic factor that captures the individual (${\mu }_{t}$ is including the permanent common component) and time-specific effects, and ${a}_{it}$, the transitory component. Philips and Sul (2007) transform (1) in a way that the common and idiosyncratic components in the panel are separated. Specifically,

4 $${X}_{it}=\left(\frac{{\mathrm{g}}_{it}+{\alpha }_{it}}{{\mu }_{it}}\right){\mu }_{it}={\delta }_{it}{\mu }_{t},for all i and t$$

where ${\mu }_{it}$  is the common factor across the states and ${\delta }_{it}$  is a time-varying idiosyncratic component that captures the individual economic performance distances between the common trend components and ${X}_{it}$.The time-varying element ${\delta }_{it}$ is modelled in a semiparametric form as:

5 $${\delta }_{it}={\delta }_{i}+{\sigma }_{it}{\varepsilon }_{it}, {\sigma }_{it}=\frac{{\sigma }_{i}}{{\mathrm{log}(t)t}^{a}},{\sigma }_{i}>0$$

where ${\delta }_{it}$ is fixed, across individual i=1, 2,…, N and weakly dependent over time t, $\alpha$ denotes the speed of convergence. Finally, L (t) is a slowly varying function, for which L (t) → ∞ as t → ∞ for $\alpha$ ≥ o.

Convergence among all states and overall convergence form the hypothesis of relevance (H_0_:$\delta$_I_ =$\delta$ *for all i with a* $\alpha$ ≥ o), against the alternative hypothesis of no convergence for a particular state or states (H_a_ :$\delta$_I_ =$\delta$ *for all i with* $\alpha$ ˂ 0). On the other hand, general divergence as well as sub-panels of states moving to various steady states or club convergence with divergent states can be observed (H_a_ :$\delta$_I_ ≠$\delta$ *for some i with* $\alpha$ *≥ 0* or $\alpha$ ˂ 0).

As ${\mu }_{it}$ represents a common element in equation (2), it can be scaled out to get the relative transition coefficient, which can assess the convergence and long-run equilibrium. ${h}_{it}$ aids in calculating the loading coefficient. ${\delta }_{it}$ represents the panel average at time t. The parameter ${h}_{it}$ can be estimated as follows:

6 $${h}_{it}=\frac{{X}_{it}}{{N}^{-1}{\sum }_{i=1}^{N}{X}_{it}}= \frac{{\delta }_{it}}{\frac{1}{N}\sum_{i=1}^{N}{\delta }_{it}}$$

In presence of convergence, there should be a common limit in the transition path of each economy and the coefficient ${h}_{it}$ should converge towards unity. if ${h}_{it} \to$ 1,${\delta }_{it} \to {\delta }_{i}$. Therefore, the variance of ${h}_{it}$ should converge towards unity, the cross-sectional variation should converge to zero when T moves towards infinity. Then we have,

7 $${H}_{it}=\frac{1}{N}\sum_{i=1}^{N}({h}_{it}-1{)}^{2} \to 0 as t\to \infty .$$

The coefficient of assessment and capture of divergent individual behaviour illustrates the relative transition route from common stochastic trends when testing the null hypothesis of convergence and grouping individuals into convergence clubs in the preceding equation. There are two components to the process. We start by determining whether or not convergence exists. Next, the potential of club convergence is investigated. The null hypothesis, according to PS, is convergence, which we evaluate using the following regression model:

8 $$log\left(\frac{{H}_{1}}{{H}_{t}}\right)-2logL\left(t\right)=\alpha +\beta logt+{\mu }_{t}$$

where for $t=\left[rT\right],\left[rT\right]+1,\dots .,T. with an r>0,$ starting with $t=\left[rT\right]$, being the integer components $rT$ for some fraction $r>0$, Phillips and Sul (58) recommend that the $r$ value be set at 0.3. Since $\beta$ *=2* $\alpha ,$ $\beta$ coefficient gives a scaled estimation of the speed of convergence parameter and under the null hypothesis the convergence parameter $\alpha$. A one-sided t test of $\alpha$ *≥ 0*, which is rejected at the 5% significance level if ${t}_{b}< -1.65$, can thus be used to test the convergence. Furthermore, $\beta$ assesses the speed of convergence of the relative transition parameter ${\delta }_{it}$ not only in the sign of the coefficient $\beta$ *=2* $\alpha$, but also in its magnitude. Hence, the estimate $\beta \ge$ *2* $,$ ($\alpha \ge 1$) denotes the absolute convergence, that is, convergence to a specific club indicates the level of convergence. This rate of convergence corresponds to conditional convergence, whereas $2\ge \beta \ge$ *0*.

In 2007, Phillips and Sul proposed a five-steps clustering algorithm that could be used to detect clubs that converge in the panel when the null of convergence in the panel is rejected. The authors of Schnurbus, Haupt, and Meier [75] recommended a few small changes to the original algorithm. The following are the main stages in order:

1. Ordering the panel members according to the last observation.
2. Form a core club in which employs the first *k* such that for the subgroup of individuals *k*, *k + 1*, the log (t) regression test statistic ${t}_{k}$ > 1.65. We may end the process and conclude that there are no convergence subgroups in the panel if there is no *k* fulfilling ${t}_{k}$ > 1.65.
3. Filter the data for new members of the core group (steps 2), which are added one at a time. In order to determine whether a convergence club has been obtained, the log (t) test is used.
4. In Step 3, run the log (t) test on all of the non-selected states. There exist convergence clubs if the t statistic is greater than 1.65. If needed, the sub-convergence clusters can be determined by repeating steps 1 through 3. If no more clubs are found, it can be considered that the other states are displaying divergent behaviour.
5. Club merging to determine final club structure: For all pairs of subsequent initial clubs, run the log (t) regression. Merge those clubs fulfilling the convergence hypothesis jointly. Schnurbus, Haupt, and Meier [75] suggested an iterative procedure for merging clubs: conduct the log t-test for the initial clubs 1 and 2; if they jointly satisfy the convergence hypothesis, merge them to form the new club 1; if not, conduct the log t-test for the initial clubs 2 and 3, etc. Then, the process can be repeated on newly obtained club classifications until no more clubs can be merged, resulting in a classification with the smallest number of convergent clubs.

### Kernel distribution estimation

Kernel density estimates are widely used in a non-parametric way to study convergence. The non-parametric estimations don’t make any assumptions regarding the normality of the data [80, 81]. As per the theoretical explanation of non-parametric estimates, the transition in mortality often happens between different states or countries with varying mortality rates [22, 76]. As countries or states reach high levels of life expectancy and low mortality, there is a convergence, causing the fading of the second peak. Therefore, it is a suitable measure of club convergence. It may be defined as:

$Let f=f(x)$ represent the continuous density function of a random variable $X$ at a given point $x, and {x}_{1},\dots ,{x}_{n}$ represent the observations from $f.$

The kernel function K can be represented as [77, 78] as follows:

9 $$\underset{-\infty }{\overset{\infty }{\int }}k\left(y\right)dy=1 where k(y)\ge 0.$$

The general kernel estimator $f^(x)$ is defined by:

10 $$\widehat{f(x)} = \frac{1}{hn}\sum_{i=1}^{n}k\left(\frac{{X}_{i}-x}{h}\right)=\frac{1}{nh}\sum_{i=1}^{n}k({y}_{i})$$

Where ${y}_{i}$ = ${h}^{-1}({x}_{i}-x)$, n defines the number of observations in the sample, and h is the window width(bandwidth), which is a function of the sample size and goes to zero as $n\to \infty$ [79].

## Data and sources

We have used the data from the Sample Registration System [36, 80] (SRS, 1990–2016, 2018) (Office of the Register General of India and Census Commissioner, 1990–2018).

The following are the various health aspects taken into account:

- Life Expectancy at Birth (LEB): Aggregate for both sexes refers to the number of years a newborn is expected to live under the current mortality rate at the time of birth. It is assumed to remain constant throughout the lifetime.
- Infant Mortality Rate (IMR): It refers to the number of infants who die before completing the first year of life per thousand live births in a given year.
- Neonatal Mortality Rate (NNMR): It refers to the number of infants dying before 28 days of life per thousand live births in a given year.
- The Total Fertility Rate (TFR): It is defined as the total number of children that would be born to each woman if she were to live at the end of her childbearing year and give birth to children in alignment with the prevailing age-specific fertility rate.

## Results

### Summary statistics of key health outcomes

We observed that the average health status in 15 major Indian states progressed steadily between 1990–2018 (Table 1). At the state level, the mean of LEB rose from 60.02 years in 1990 to 69.94 years in 2016, implying that the states have made significant progress in life expectancy. According to the association between gains in life expectancy at birth and the baseline level (1990), the states with lower baseline levels have improved more than those with higher baseline levels. The difference between the advanced and poorest performing states concerning life expectancy at birth has been narrowing gradually.

**Table 1:** Summary statistics of key health outcomes

Year	Mean	SD	Cov	Min	Max	Range
**Life Expectancy at Birth (LEB)** (in Years)						
1990	60.02	4.85	0.081	53.4	70.9	17.5
2000	63.75	3.79	0.059	58.0	71.9	13.9
2010	67.59	3.25	0.048	62.7	74.7	12.0
2016	69.94	2.61	0.037	65.3	75.3	10.0
**Infant Mortality Rate (IMR)**^a^						
1990	73.73	24.62	0.334	17	122	105
2000	63.20	19.81	0.313	14	95	81
2010	43.40	15.05	0.347	13	62	49
2018	28.93	11.47	0.397	7	48	41
**Neonatal Mortality Rate (NNMR)**^b^						
1990	48.19	15.95	0.331	12.6	78.8	66.2
2000	41.13	12.92	0.314	9.8	61.1	51.3
2010	29.59	10.36	0.350	7.1	44.2	37.1
2018	20.33	8.50	0.418	5.0	35.0	30.0
**Total Fertility Rate (TFR)**^c^						
1990	3.58	0.915	0.256	1.9	5.2	3.3
2000	3.02	0.898	0.297	1.9	4.7	2.8
2010	2.39	0.677	0.283	1.7	3.7	2.0
2018	2.07	0.535	0.258	1.5	3.2	1.7

*Note*: *SD* Standard deviation, *COV* Coefficient variation, *Min* Minimum, *Max* Maximum, *N* 15

*Source*: Author’s estimation from Sample Registration System (SRS), India (1990-2018)

^a^the number of infant deaths per 1,000 live births

^b^the number of neonatal deaths per 1,000 live births

^c^the average number of children that would be born to a woman over her lifetime

In contrast, IMR and NNMR mortality rates have dramatically dropped from an average of 73.73 and 48.19 deaths per 1000 live births in 1990 to a record of 28.93 and 20.33 deaths per 1000 live births, respectively, in 2018 (Table 1). The IMR and NNMR in 1990 ranged from 122 and 78.8 to a minimum of 17 and 12.6 deaths per 1000 live births, respectively. In 2018, it went from 48 and 35 to 7 and 5, respectively. The lagged regions with high infant and neonatal mortality levels in the initial period have significantly reduced more than the advanced regions. The disparity between advanced and low-performing states in IMR and NNMR has been increasingly shrinking.

The total fertility rate is assessed in Table 1 using a similar procedure, and the results indicate that, on average, the TFR has decreased from a high of 3.58 in 1990 to a low of 2.07 in 2018. The TFR in 1990 ranged between a maximum of 5.2 and a minimum of 1.9. It varied from 3.2 to a minimum of 1.5 in 2018. The lagging states with a high beginning total fertility rate have significantly declined compared to the developed regions at the initial TFR level. The TFR disparity between advanced and lag-performing states has steadily diminished over time (from 3.3 in 1990 to 1.7 in 2018). However, total fertility rates are declining, and the disparity across states has widened significantly throughout the sample period.

### Sigma convergence

**Fig. 1:**
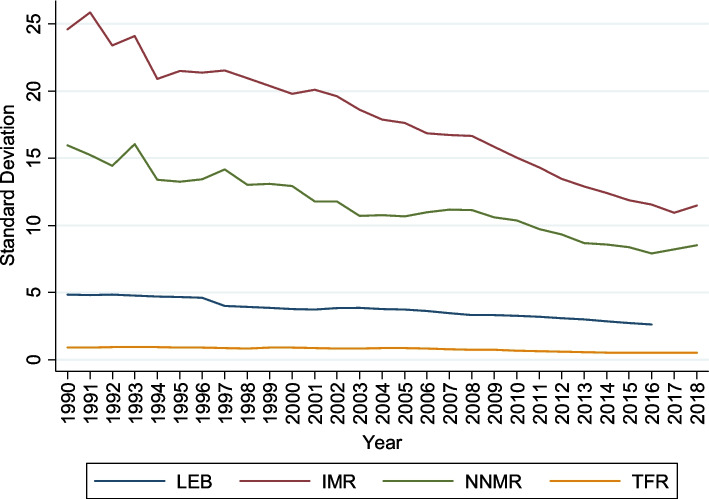
The σ Convergence of Selected Key Health Indicators

Figure 1 shows the sigma convergence Quah, [79] measured by analyzing the progress in (σ) standard deviation of the selected health indicators (LEB, IMR, NNMR, TFR) across the major states. The results indicate that the standard deviation for the selected health indicators shows a constant decline in variation across different groups during 1990–2018. There is clear evidence for convergence of the health outcome, and the convergence process is underway in almost all the selected health indicators.

**Fig. 2:**
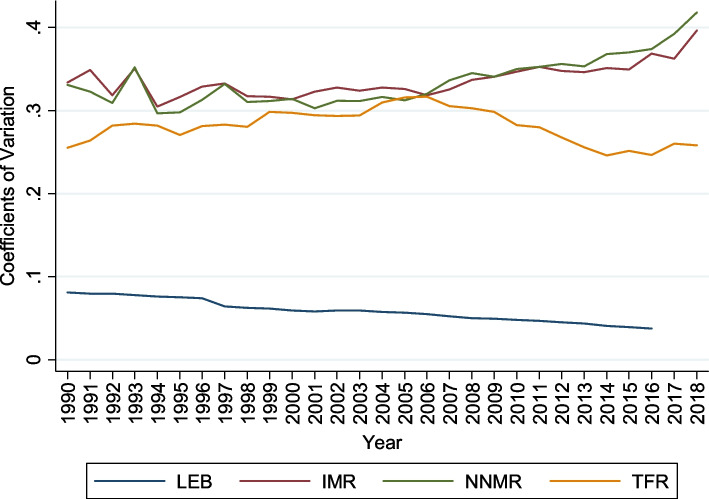
Coefficient Variation of Selected Key Health Indicators

Figure 2 shows the coefficient variation (CV) decline for selected health indicators (LEB, IMR, NNMR, and TFR). It denotes a reduction in the cross-sectional dispersion in health status over the sample period from 1990 to 2018. The convergence pattern is more evident for life expectancy at birth. For IMR and NNMR, the CV declined rapidly between 1990 and 2006, then gradually increased. The CV for TFR quickly increased between 1990 and 2006, after which there has been a gradual decline of around 0.3 to 0.2 CV, narrowing the disparity across states. These findings show that the Indian states have different patterns; therefore, the club convergence study will provide valuable information about their grouping. Our primary concern is the dynamic pattern of club convergence of selected health indicators across the states.

### Club convergence: log t-test

The results are obtained by applying the Phillips and Sul (58) [64] methodology to selected health indicators to identify the potential convergence and divergence patterns among the major Indian states.

#### Convergence in LEB

**Fig. 3:**
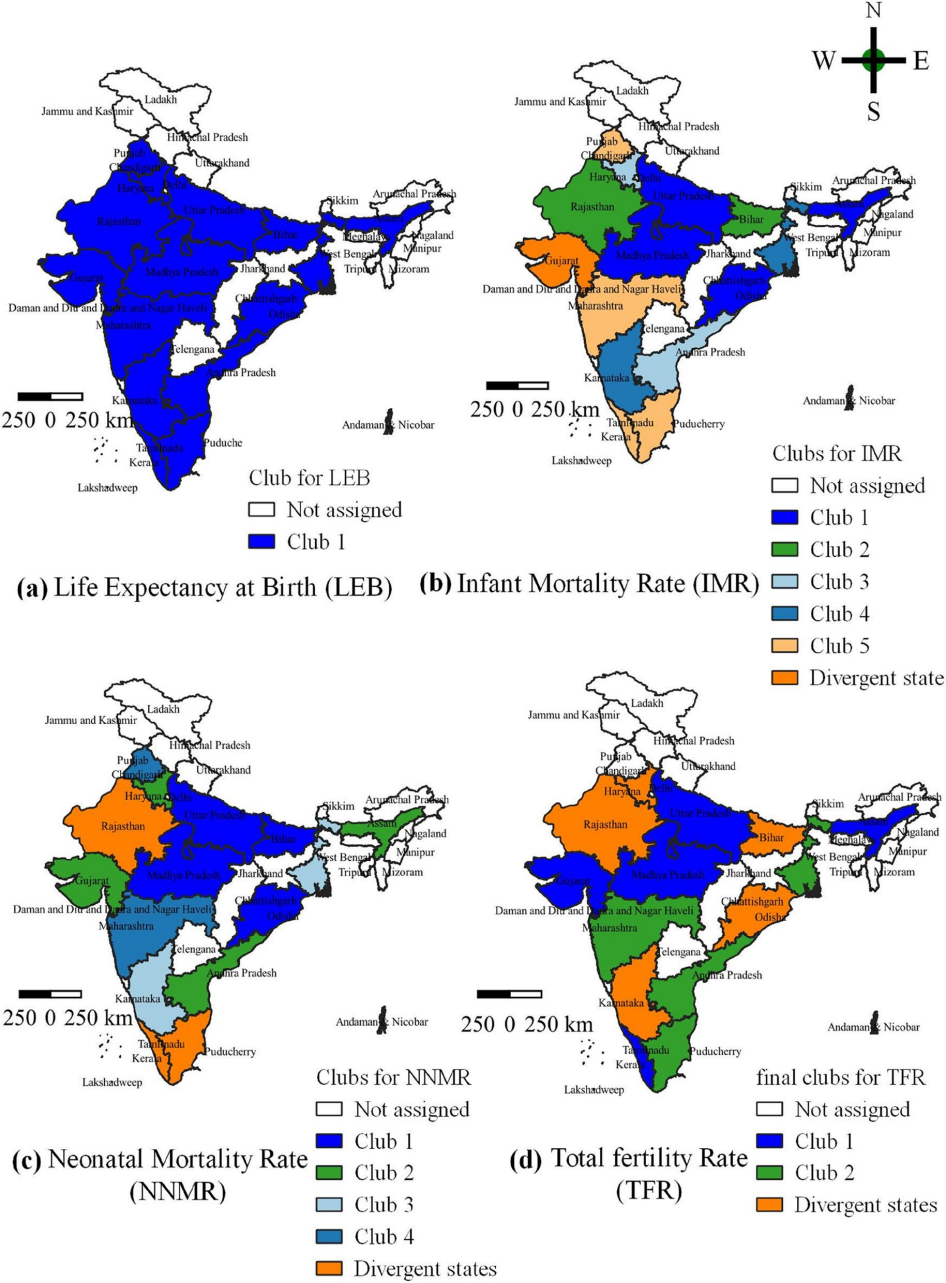
Estimated Clubs for the Key Health Outcomes (**a**) LEB. **b** IMR. **c** NNMR. **d** TFR. *Source*: Authors’ compilation based on data from the Sample Registration System (SRS). The map was developed by the authors using QGIS Version 3.24.0, and the map was cross verified with the India map and its States and Union Territories’ boundaries as shown on the official website of the Survey of India: https://indiamaps.gov.in/soiapp/

In the total sample of LEB, the log (t) test of the value is 3.93, greater than the critical value − 1.65; thus, we do not reject the null hypothesis of convergence (Table 2). This result suggests an absence of a club convergence transition path but reveals a unique transition path among the states. The convergence study of LEB shows that all states belong to club one, indicating that LEB is converging in the same direction in all states (Table 2). Figure 3a depicts an apparent geographical reference to India’s major states. The presence of the LEB is a common feature of the Indian states in the first club.

**Table 2:** Test for convergence in key health outcomes

Initial classification	States	Coef	log(t)-stat	Inference
**Life Expectancy at Birth (LEB) 1990-2016**				
Full samples [15]		0.2936	3.9343	Convergence
Club 1 [15]	Andhra Pradesh, Assam, Bihar, Gujarat, Haryana Karnataka, Kerala, Madhya Pradesh, Maharashtra, Odisha, Punjab, Rajasthan, Tamil Nadu, Uttar Pradesh, West Bengal	0.344	6.733	
**Infant Mortality Rate (IMR) 1990-2018**				
Full samples [15]		-0.9935	-38.952^a^	Divergence
Club 1 [4]	Assam, Madhya Pradesh, Odisha, Uttar Pradesh	0.792	2.718	Club convergence
Club 2 [2]	Bihar, Rajasthan	0.765	7.799	Club convergence
Club 3 [2]	Andhra Pradesh, Haryana	2.457	3.097	Club convergence
Club 4 [2]	Karnataka, West Bengal	1.963	2.757	Club convergence
Club 5 [4]	Punjab, Maharashtra, Kerala, Tamil Nadu	0.0251	3.172	Club convergence
Group 6 [1]	Gujarat	-0.472	-10.649	Divergence state
**Merge of Clubs**				
Club 1+2 [6]		-0.3983	-4.8465	No merge
Club 2+3 [4]		-0.92	-13.244	No merge
Club 3+4 [4]		-1.575	-19.817	No merge
Club 4+ 5 [6]		-0.14	-3.533	No merge
Club 5+ 6 Group [5]		-0.282	-8.6842	No merge
**Neonatal Mortality Rate (NNMR) 1990-2018**				
Full samples [15]		-1.3637	-10.4607^a^	Divergence
Club 1 [4]	Bihar, Odisha, Madhya Pradesh, Uttar Pradesh	0.221	2.1	Club convergence
Club 2 [4]	Assam, Haryana, Gujarat, Andhra Pradesh	1.701	3.693	Club convergence
Club 3 [2]	Karnataka, West Bengal	2.435	11.562	Club convergence
Club 4 [2]	Maharashtra, Punjab	1.263	0.681	Club convergence
Group 5[3]	Rajasthan, Kerala, Tamil Nadu	-1.13	-62.486	Divergence states
**Merge of Clubs**				
Club 1+2 [8]		-1.2314	-27.17	No merge
Club 2+3 [6]		-1.3822	-107.17	No merge
Club 3+4 [4]		-2.8253	-8.416	No merge
Club 4+ Group 5 [5]		-1.1132	-58.5656	No merge
**Total Fertility Rate (TFR) 1990-2018**				
Full samples [15]		-1.1036	-10.4607^a^	Divergence
Club 1 [3]	Madhya Pradesh, Uttar Pradesh, Kerala	0.097	1.663	Club convergence
Club 2 [2]	Assam, Gujarat	0.086	0.496	Club convergence
Club 3 [5]	Andhra Pradesh, West Bengal, Tamil Nadu, Punjab, Maharashtra	0.314	2.319	Club convergence
Group 4 [5]	Bihar, Haryana, Karnataka, Odisha, Rajasthan	-1.451	-254.099	Divergence states
**Merge of Clubs**				
Club 1+2 [5]		-0.0347	-0.6743	Merge
Club 2+3 [7]		-1.7713	-150.126	No merge
Club 3+Group 4 [10]		-1.5345	-186.465	No merge
**Final club for TFR**		After merged		
Club 1 [5]	Assam, Gujarat, Kerala, Madhya Pradesh, Uttar Pradesh	-0.035	-0.647	Club convergence
Club 2 [5]	Andhra Pradesh, Maharashtra, Punjab, West Bengal, Tamil Nadu	0.314	2.319	Club convergence
Group 3 [5]	Bihar, Haryana, Karnataka, Odisha, Rajasthan	-1.451	-254.099	Divergence states

*Note*: The critical value is -1.65 at 5% level of significance level

*Source*: Author’s estimation from Sample Registration System (SRS)

^a^Indicates rejection of null of convergence. Inside the [] indicates the number of states are calculated in the box

#### Convergence in IMR

The results of the club convergence approach in IMR are reported in Table 2. Considering the entire sample, the log (t) value is -38.952, less than the critical value of -1.65. Thus, we reject the null hypothesis of convergence at the 5% significance level. The results indicate that the IMR across states does not converge on a single transition path. Hence, clubs may employ the PS clustering algorithm. The evidence indicates that fifteen states out of five clubs with four, two, two, two, and four states, respectively, are statistically significant, and the only one state that does not converge with any of these states (Table 2). These clubs' log (t) values are 2.718, 7.799, 3.097, 2.757, and 3.172, respectively. Each value is higher than the critical value (i.e., -1.65). Therefore, we are unable to reject the null hypothesis. The PS clustering algorithm’s implementation among the clubs dictates whether smaller clubs may be merged into larger ones. The findings further indicate that merging clubs 1 + 2, 2 + 3, 3 + 4, and 4 + 5 are considerably convergent, with log (t) values of -4.8465, -13.244, -19.817, and − 3.5329, respectively; however, club 5 + group 6 (Divergent state) does not converge, with a log (t) value of -8.6842 (Table 2). Table 2 shows that infant mortality rates represent a model of convergence by showing the convergence club in states with different infant mortality rates. We found that five clubs exhibit patterns of convergence, and Gujarat is an exception, as it exhibits divergence in infant mortality rates (Table 2). The infant mortality rate in each club is shown in Fig. 3. In Fig. 3b IMR, the states that make up these clubs are divided geographically, with the various clubs represented by various colors.

#### Convergence in NNMR

The outcome of convergence in NNMR is shown in Table 2. The estimated value of log (t) is -10.6455 (< -1.65). Since the log (t) value is below the critical value, we can reject the null convergence hypothesis at the 5% significance level. The result suggests the existence of a convergence club, which might contribute to the emergence of an algorithm for detecting NNMR. According to the convergence estimates in Table 2, clubs 1 and 2 include four states each, clubs 3 and 4 contain two states, and group six has three non-convergent states (Table 2). These clubs ‘log (t) values are 2.1, 3.693, 11.562, and 0.681, respectively. Each value is higher than the critical value (-1.65). Implementing the PS clustering algorithm among the clubs determines whether smaller clubs can be merged into larger ones. The findings in Table 2 indicate that the merging clubs 1 + 2, 2 + 3, and 3 + 4 converge strongly with log (t) values of -27.17, -107.17, and − 8.416, respectively; however, club 4 + group 5 (Divergent states) does not converge with a log (t) value of -58.5656. The progression of the NNMR for the various estimated clubs is seen in Table 2. The progress of the NNMR for the various estimated clubs can be seen in Fig. 3. The Neonatal Mortality Rate of Clubs 1–4 is presented in Fig. 3c, and three states—Kerala, Rajasthan, and Tamil Nadu—show divergence.

#### Convergence in TFR

The analysis reveals that the log (t) statistic value is -10.64, less than the crucial threshold (-1.65). We can reject the null hypothesis of convergence of TFR at the 5% significance level (Table 2). The result implies that TFR between states does not always converge on a path along which a club may exist. Three clubs are identified to identify them employing the algorithm approach. Club 1 consists of three states, club two consists of two states, club three consists of five states and Group 4 consists of five divergent states (Table 2). These clubs' log (t) values are 1.663, 0.496, and 2.319, respectively (Table 2). Each value is higher than the critical value (-1.65) – thus, we cannot reject the null hypothesis. Furthermore, Phillips and Sul (58) suggest that their technique exaggerates rather than underestimates club convergence. The clustering approach examines the evidence for clubs merging into bigger or between clubs. After analyzing the pattern of the final club, we concluded that each state has its particular way of clubbing. The result indicates that the merger of club 1 + 2 with a log (t) of -0.6743 is not significant but that the merger of club 2 + 3 with a log (t) of -187.98 is significant (Table 2). However, the merger results reveal the two final convergence clubs and one divergence group in TFR activities. Club 1 and 2 have five states with log (t) values of -0.647 and 2.319, respectively (Table 2).

Additionally, group 3 comprises five states, including Bihar, Haryana, Karnataka, Odisha, and Rajasthan, which need to be convergent. The evolution of the TFR for the various estimated clubs is seen in Table 2. As seen in Fig. 3, a distinct geographical separation exists between the states represented in these clubs. The area is easily divided into two final clubs and a divergence group (Divergent states). The final club's total fertility rate distribution is shown in Fig. 3d. As can be seen, five states belong to each of the two groups, and Bihar, Haryana, Karnataka, Odisha, and Rajasthan exhibit a similar diverging trend

#### Club convergence: kernel density plots

**Fig. 4:**
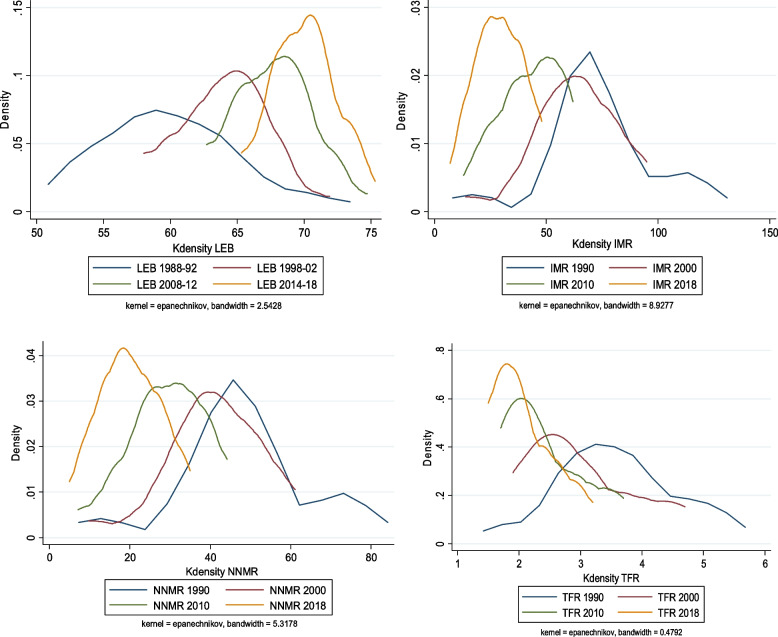
Kernel Density Distribution of select health Indicators. Kernel Density Distribution of Selected Key Health Indicators

The findings from testing the hypothesis of convergence clubs through kernel density plots indicate the presence of stratification, polarization, and clubs’ convergence of selected health indicators in major Indian states over the study period (Fig. 4).

The distribution of life expectancy at birth is widely spread during the initial period (1988-92) compared to recent years (2014-18). According to kernel density estimates, life expectancy at birth stratifies, polarizes, and becomes unimodal through time, although with a single stable state. In recent years, the maximum number of states have exhibited a higher level of LEB, indicating the emerging convergence process of LEB across major Indian states. In the case of IMR and NNMR, there was a wider spread in 1990 compared to 2018, which portrays a larger peak. The result indicates a larger peak at lower levels of IMR, NNMR, and a smaller secondary peak with higher IMR, NNMR. The kernel plots show stratification’s presence, polarized and bimodal over time. This phenomenon indicates the presence of a convergence “club” in 2018. Siddiqui et al., [51] analyzed the current club convergence of health indicators across Indian states, and our analysis aligns with theirs. In the case of TFR, the highest peak with a greater value of states is observed in the initial period (1990), whereas in the recent period, two widely spread peaks can be observed. Kernel density plots in TFR show that the early phase is stratified, but the distribution of states becomes polarized and bimodal over time. The figure suggests that most states’ fertility rate was higher compared to the recent period, indicating the existence of a convergence club among the major Indian states.

## Discussion

Health affects the economy in explicit and implicit ways. Therefore, evaluating the various health aspects is imperative to understand and work toward economic development. The convergence theory in health progress identifies gaps in current health policies and strongly emphasizes inclusive and strategy-oriented approaches. The present study employs the sigma convergence, log t-test, and kernel density estimator to identify patterns of convergence, divergence, and club convergence in selected health outcomes, namely, life expectancy at birth, infant mortality rate, neonatal mortality rate, and total fertility rate for 15 major states of India since 1990.

The study’s outcomes reveal that all the states have shown remarkable improvement in life expectancy at birth. However, states with high performance in life expectancy at birth have become stagnant, with little progress in improving life expectancy at birth. In contrast, a faster improvement can be observed in the states with lower life expectancy at birth. The lagged states have converged in life expectancy at birth and are catching up with the better-performing states. Our findings corroborate the sigma convergence analysis of life expectancy at birth (LEB) across states throughout the period. The study demonstrates the prevalence of convergence over the whole sample. The kernel density estimates illustrate how they converge to a steady state, as shown by the unimodal value of LEB. A rapid improvement in life expectancy at birth at regional, state, or country levels will contribute substantially to economic development [29, 54, 81–83]. Despite the improvement in life expectancy at birth, at regional levels, the progress rate has been slow recently.

However, the outcome was somewhat different. Intriguingly, the results of sigma convergence in IMR, NNMR, and TFR indicated convergence between 1990 and 2006 but sigma divergence afterward. Other findings, such as the kernel density estimator distribution, bolstered the notion of a convergence club by demonstrating the existence of a bimodal distribution for all IMR, NNMR, and TFR indicators. Additionally, the log t-test findings corroborate the occurrence of club convergence and heterogeneity in the overall health indicator analysis.

The Convergence Club is found in states with varying infant mortality rates, demonstrating that infant mortality rates reflect a convergence model. We found that five clubs exhibit convergence patterns, and one state depicts divergence. Club One is made up of four states: Assam, Odisha, Madhya Pradesh, and Uttar Pradesh, whereas club two is made up of two states: Bihar and Rajasthan. Club Three comprises the states of Andhra Pradesh and Haryana, club four comprises the states of Karnataka and West Bengal, and club five comprises the states of Kerala, Punjab, Maharashtra, and Tamil Nadu. However, Gujarat is an exception, as it exhibits divergence in infant mortality rate. Therefore, there is a need for Gujarat to accelerate the process of reduction in infant mortality rate and to join the convergence process. This view is concurrent with the existing literature, which indicates that regional inequality within states and divergent progress within lagging states harm overall progress [48, 55, 84].

The neonatal mortality rate also varies significantly between the major Indian states' clubs. The finding indicates a lack of unique convergence among states, implying that club convergence and divergence occur across states. Bihar, Odisha, Uttar Pradesh, and Madhya Pradesh exhibit convergence in club one. Similarly, club two includes Assam, Haryana, Gujarat, and Andhra Pradesh; club three - Karnataka and West Bengal; and club four - Maharashtra and Punjab. On the other hand, Kerala, Rajasthan, and Tamil Nadu have demonstrated divergence. The result reflects progress in the neonatal mortality rate through the convergence of clubs. However, the divergence among states reflects increased state variation and disparity in neonatal mortality rates. Some clubs have accelerated their progress similarly, whereas a few have remained virtually stagnant and have shown divergence. The results align with the findings of earlier research studies based on child and neonatal mortality [51, 54, 85]. The findings suggest that states should continue monitoring effective health interventions to reduce neonatal mortality and variation among the states.

Further, the finding of the inequality-based convergence measure on the total fertility rate demonstrates the existence of convergence clubs and divergence across major states. Club one comprises five states: Assam, Gujarat, Kerala, Madhya Pradesh, and Uttar Pradesh, whereas club two comprises states Andhra Pradesh, Maharashtra, Punjab, West Bengal, and Tamil Nadu. On the other hand, Bihar, Haryana, Karnataka, Odisha, and Rajasthan, have shown divergence. Convergence occurs when the difference between the state variations declines [27]. States that initially had a high fertility rate are catching up with those with a low fertility rate. However, some states have a diverging fertility pattern and should continue following the convergence process [44]. Each club with a total fertility rate difference implements strategies to reduce the variance between India's major states.

This analysis shows that India’s trends in average health status, particularly the increase in life expectancy at birth, the decrease in infant and neonatal mortality rates, and total fertility, all indicate the country's momentum toward growth and development. India has enacted diverse health policies to tackle many health issues and enhance its populace’s overall welfare. These policies have targeted vital indicators such as improving life expectancy at birth, reducing child mortality rates, and controlling total fertility rates. Some notable health policies in India include the National Health Policy (2017), which delineates the government’s strategic framework for attaining universal healthcare coverage and enhancing the general well-being of the Indian population. It emphasizes the importance of bolstering primary healthcare, enhancing public health expenditure, and improving the accessibility of high-quality healthcare services [86]. The National Rural Health Mission (NRHM) focused on how to enhance the efficacy of primary healthcare services by implementing community-driven public health interventions at the grassroots level. This approach aimed to mitigate disparities in healthcare accessibility and reduce child mortality rates [87]. Ayushman Bharat- Pradhan Mantri Jan Arogya Yojana (PM-JAY) aimed to reduce the reduce the out of pocket expenditure while availing health care services and to converge various health insurance schemes across states [88]. Janani Suraksha Yojana (JSY), By providing financial incentives to pregnant women who choose to give birth at healthcare facilities, JSY promotes institutional deliveries. This effort seeks to minimize maternal and neonatal mortality by ensuring safe deliveries and skilled medical care during childbirth [89]. Pradhan Mantri Swasthya Suraksha Yojana (PMSSY): This also has affordable and reliable tertiary-level healthcare in the country and augments facilities for quality medical education in the under-served State [90]. National population policy and recognizing Indian Systems strategy aimed to lower fertility to replacement level by 2010 [91]. The government acknowledged the significance of Indian medicine, homeopathy in healthcare, and many more health policies. Government policies, such as the abovementioned, are crucial in establishing efficient integration and convergence of life expectancy at birth. However, it is essential to note that these policies encounter challenges regarding inadequate investment in reducing child mortality and controlling the total fertility rate, particularly in states that need to catch up.

However, improving health status and achieving convergence across states can only ensure progress and stability if effective health intervention measures are implemented, particularly in divergent states that reflect the variation. The results show that club convergence and divergence exist among the states across the various health dimensions such as IMR, NNMR, and TFR. Several states in India have devised and implemented specific health policies and programs aimed at mitigating child mortality and controlling the total fertility rate. The interventions above consider challenges and healthcare needs that vary between club convergence and divergence across states. Prior studies have indicated significant disparities in socio-economic conditions, diverse socio-economic progress, healthcare spending, sectoral distribution, and policy environments are crucial in shaping divergent health outcomes such as IMR, NNMR, and TFR [29, 51, 54, 92]. Moreover, policies should allocate resources to critical healthcare interventions. Comprehensive frameworks are necessary for cost-effective healthcare measures, enhanced investments, and improved technology access. Increased funding for health research and the availability of reliable and complete data on diseases that disproportionately affect states with lower performance and divergence are also essential.

The interstate health disparity is an obstacle to overall progress and development. The gap between the clubs and divergent states in IMR, NNMR, and TFR must be reduced to ensure optimal growth and development. Therefore, formulating strategies to eradicate IMR and NNMR is crucial for achieving progress, change, and convergence across the states. A robust program of action and policies focused on achieving equity with efficiency needs to be formulated. The Government of India can incorporate the study’s findings to formulate policies to establish effective integration to achieve equity in health status among various states. Convergence analysis is crucial for achieving the SDGs at the regional, national, and global scales.

## Conclusion

This study explored the regional convergence of selected health outcomes in fifteen major Indian states from 1990 to 2018. Results of the sigma convergence model reveal that life expectancy at birth has increased steadily, except for infant mortality, neonatal mortality, and total fertility rates, which have diverged. Club convergence analysis was used to elucidate these findings. The findings demonstrate significant regional convergence in many health indices, most notably life expectancy at birth, emphasizing state-level club convergence and divergence in infant mortality, neonatal mortality, and total fertility rates. The kernel density estimates indicate that life expectancy at birth follows a cyclical pattern with a unimodal characteristic classified endogenously, albeit with a common steady state. However, the research bolstered the convergence club concept by confirming the presence of a bimodal distribution for some health variables, including infant mortality, neonatal mortality, and total fertility. Health efforts should focus on lowering high death rates and improving survival rates in all the states. The report emphasizes the need to concentrate on health-related issues and close the gap between advanced and lagged states. This is essential for promoting health equity and contributing to development. As a result, we must address regional development equity and balance, especially regarding health outcomes. Further research is necessary to explore the causal link mechanism in greater depth, as it poses a notable limitation in the present study. Our analysis sheds insight into the health equity policy decisions made by Indian states.

## Data Availability

The datasets generated and/or analyzed during the current study are publicly available in the Sample Registration System (SRS), https://censusindia.gov.in/census.website/, which is supported and maintained by the Office of the Registrar General & Census Commissioner, India (ORGI).
